# Framing the numerical findings of Cochrane plain language summaries: two randomized controlled trials

**DOI:** 10.1186/s12874-020-00990-4

**Published:** 2020-05-06

**Authors:** Ivan Buljan, Ružica Tokalić, Marija Roguljić, Irena Zakarija-Grković, Davorka Vrdoljak, Petra Milić, Livia Puljak, Ana Marušić

**Affiliations:** 1 Department of Research in Biomedicine and Health, University of Split School of Medicine, Šoltanska 2, 21000 Split, Croatia; 2 Cochrane Croatia, University of Split School of Medicine, Split, Croatia; 3 Department of Oral Diseases and Periodontology, University of Split School of Medicine, Split, Croatia; 4 Department of Family Medicine, University of Split School of Medicine, Split, Croatia; 5 University of Split School of Medicine, Split, Croatia; 6 Center for Evidence-Based Medicine and Health Care, Catholic University of Croatia, Zagreb, Croatia

**Keywords:** Plain language summaries, Information translation, Evidence summaries, Health numeracy

## Abstract

**Background:**

Cochrane systematic review Plain language Summaries (CSR PLSs should serve as a tool for the evidence translation to non-medical population. However, the evidence of optimal type of numerical presentation in CSR PLSs is still scarce. The aim of this study was to investigate readers’ comprehension and preferences for different presentation of findings, including framing and numerical data, in Cochrane systematic review Plain Language Summaries (CSR PLSs).

**Methods:**

We conducted a parallel randomized trial and a crossover randomized trial at the School of Medicine and family practice offices in Split, Croatia. The participants were students and consumers. We assessed possible differences in comprehension, measured by four questions on PLS content, of CSR PLSs depending on the positive or negative framing of results (*n* = 91) (Trial 1) or using percentages or frequencies for the presentation of results (*n* = 245) (Trial 2). The outcome measures were comprehension of PLS content, perceived effectiveness of the treatment and readiness to use the treatment (all on 1–10 scales).

**Results:**

In Trial 1 we found no difference in readers’ perception of the effectiveness of the described treatment, desire that the treatment be offered by their family doctor, readiness to use the treatment, or comprehension when CSR PLS results were presented positively or negatively. In Trial 2 we found no difference in CSR PLS comprehension when results were presented as natural frequencies or percentages (*BF*_*10*_ = 0.62, Bayesian t-test for independent samples).

**Conclusions:**

Numerical presentation and framing direction of results appear to have no significant impact on understanding of messages in CSR PLSs.

**Trial registration:**

The trials were registered in ClinicalTrials.gov. Protocol registration numbers: Trial 1: NCT03442387; Trial 2: NCT03554252.

## Background

Recommendations for presenting health information to consumers include short formats, framing the results in a positive direction, using plain language, and situating the results in context relevant for lay audiences [1]. Studies that identify optimal formats for information presentation to consumers are important for organizations that are involved in the translation of health information to the public. One such organization is Cochrane, which focuses on producing high quality evidence about health in the form of Cochrane systematic reviews (CSRs). Cochrane undertakes large efforts to present the evidence to the lay public in formats that are acceptable, easily accessible and comprehensible [2]. These include Plain Language Summaries (PLSs) – brief summaries of systematic reviews written in plain language, and infographics – visual presentations accompanied with simple text. Despite standards and writing recommendations, it has been shown recently that Cochrane systematic review PLSs (CSR PLSs) were very diverse, varying in size and structure [3], and often including non-plain language, possibly making the understanding of the content harder for readers.

Recent research efforts have focused on health numeracy – the concept that describes a consumers’ ability to understand numerical expressions and perform calculations in a health context. Numeracy has been shown as an important predictor of various health conditions and outcomes [4]. In that sense, individuals with low levels of health numeracy are at risk, because failing to understand health information leads to poor treatment outcomes [5]. Therefore, it is important to tailor health messages in a way that makes them understandable to individuals with low health numeracy levels. However, previous research has indicated that issues with framing of health information are not restricted to patient populations only, but that physicians also have issues with the numerical framing of information [6]. Cochrane’s Plain Language Expectations for Authors of Cochrane Summaries (PLEACS) standards recommend that it is not essential to provide numerical information in PLSs, but if there are numbers presented, the presentation should be consistent, comprehensive to the lay population in terms of absolute effects, and framed as natural frequencies [7]. However, a systematic review on framing effects in health messages could not identify studies on how framing affects the comprehension of health information or on the comprehension of numerical formats in Cochrane plain language summaries [8]. Therefore, the aim of this study was to explore how different framing of numerical information influences understanding of information in CSR PLSs.

## Methods

### Development of PLSs

We conducted two randomized controlled trials (RCTs) in which we used evidence summaries, in the form of PLSs, as interventions. We used PLSs from five CSRs that addressed common health issues and would be of general interest to consumers [9–13]. All PLSs were written in a structured format, contained numbers about treatment benefits and side effects, and had a similar amount of text. For the purpose of our trials, we modified the PLSs to reflect the trial’s intervention. In order to standardize the language characteristics of the formats, all summaries were first checked using the IBM Watson Tone Analyzer [14] and then the text was refined to ensure similarity in emotional tone and sentiment, so that each summary had similar contents of three emotional tones: sadness, analytic and tentativeness (total – over 50% for the three tones). PLSs were also standardized for structure, so that after modification all PLSs were under 500 words long, consisted of four paragraphs entitled: “What is this (review) about?”, “Why is it important?”, “What evidence did we find?” and “What is the quality of evidence?” for Trial 1 and “What is this (review) about?”, “What did researchers do?”, “What evidence did researchers find?” and “What is the quality of evidence?” for Trial 2. PLSs were then translated into Croatian and back translated by a professional translator to assure the validity of the translation. There were no significant changes after back translation.

For the purpose of the first trial, we additionally customized PLSs to contain positive or negative framing of health information.

The interventions were presented in a questionnaire, which had three parts in both trials – demographic data, questions about the PLSs, and a numeracy test. They were delivered in pen and paper format and all the materials used in Trials 1 & 2 are presented in the Supplement.

Trial 1: Positive vs negative framing of health evidence.

#### Study design, setting and participants

This was a two-arm, double blind, parallel randomized trial, conducted at the University of Split School of Medicine, Split, Croatia in January 2018. First-year medical students were invited to participate. Participation was voluntary and anonymous. Each participant received one questionnaire format, which they put in a sealed envelope after completion to ensure anonymity. There was no time restriction, and the use of calculators, cell phones and the Internet was not allowed.

#### Intervention

In the positively-framed (intervention) group, the results were framed in such a way that they presented the therapy in terms of effectiveness (e.g. “The treatment was effective for 4 out of 10 people.”), while in the negatively-framed (control) group the results were framed in terms of ineffectiveness (e.g. “The treatment was ineffective for 6 out of 10 people.”). Each participant was presented with three different PLSs, all framed in the same direction in a trial arm to control for different PLS topics.

#### Randomization

Surveys were sorted in the order generated by an online software (https://www.randomizer.org/). To ensure allocation concealment, surveys were placed in sequentially numbered opaque sealed envelopes, which were distributed to the participants.

#### Primary outcomes

After reading each PLS, participants were asked to provide their assessment of the following:

1. The perceived effectiveness of the described treatment,
2. Their desire that the described treatment be offered by their family doctor,
3. Their readiness to use the treatment themselves or by a family member.

Each statement was assessed on a Likert-type scale ranging from 1 – “do not agree at all” to 10 – “fully agree”. The results were expressed as the sum of assessments of all three PLSs (total score range 3 to 30).

#### Secondary outcome

Comprehension of the content of the summary format was assessed by a brief knowledge test with four multiple choice questions for each PLS (one correct answer out of 4 offered; total possible test score = 12). The questions were pilot tested for face validity with 3 experts before the trial.

#### Blinding

The participants were blinded to the study design and randomization and monitored by their teachers during the study to prevent communication among themselves. The researchers who distributed the surveys were different from the researchers who randomized and prepared the questionnaires. They were asked to participate in a survey about the presentation of health information. The questionnaires had the same first page, regardless of the trial group and distributors were asked to distribute them to the next participant from the top of the survey package they received. Students completed the questionnaires before the start of their lectures. The survey took around 20 min to complete.

#### Sample size

We calculated the sample size based on data from research of attribute framing [15] (M_diff_ (mean difference) =1.1 on a 1–6 scale). We used sample size calculator (MedCalc Statistical Software version 17.6, Ostend, Belgium) with an alpha of 0.05 and 80% power to estimate a sample size of 12 participants in each group (24 in total) to detect a 20% difference in average scores of evaluation of treatment effect on a scale from 1 to 10 (M_diff_ = 2.00, SD_1_ = 1.17, SD_2_ = 2.01) between format groups.

Trial 2: Comparison of numerical presentation: frequencies vs percentages.

#### Study design, setting and participants

We performed a randomized, two-arm double blind trial, with a crossover study design conducted simultaneously at the University of Split School of Medicine, in a family practice in Split, in a family practice on the island of Brač, and at the University Hospital of Split. The participants at the medical school were second- to fifth-year pharmacy students, first- and second-year dentistry students and third-year medical students, whereas eligible participants at the hospital and family practices were patients ≥18 years of age.

#### Interventions

The type of numerical presentation of the results (frequencies or percentages) in a PLS represented the interventions. The task for the participants was to read two PLSs and answer questions about their content. In half of the surveys (Group A), the first PLS presented treatment effectiveness as natural frequencies (e.g. the treatment was effective for 4 out of 10 people) and side effects as percentages (e.g. 20% of the people experienced side effects). In the second PLS, treatment effectiveness was presented as percentages (e.g., the treatment was effective for 40% of the participants) and side effects as natural frequencies (e.g. 2 out of 10 people experienced side effects). Group B received a survey where the first PLS presented the results as percentages and side effects as frequencies, and the second PLS had a reverse presentation. In this way, each participant read two PLSs with the same health information but with different combinations of numerical formats, in different order. In the total of 8 possible numerical phrases in two PLSs presented to the participant, 4 were expressions presented as natural frequencies and 4 were presented as percentages.

#### Primary outcome

Comprehension of the content of summary formats was assessed by a brief knowledge test with four multiple choice questions for each PLS, 2 focusing on the results and 2 on the side effects (one correct answer out of 3 offered; total possible test score = 2 per PLS). The task for the participants was to choose a correct answer about the effectiveness of the described treatment, but the answers were always presented in the opposite numerical format than contained in the summary to examine whether the participants could relate the results presented in frequencies to percentages in the questions and vice versa. The final score was calculated as the sum of correct answers (score range = 0 to 2).

#### Secondary outcomes

After reading the PLSs, the participants were asked to provide their assessment of the following:

1. Their preference for this type of presentation of health information,
2. The effectiveness of the described treatment for the described medical issue.

Each statement was assessed on a Likert-type scale ranging from 1 – “do not agree at all” to 10 – “fully agree”.

#### Randomization

Randomization was conducted using online software (https://www.randomizer.org/). For allocation concealment, questionnaires were placed in sequentially numbered opaque sealed envelopes according to the randomization order, so that each participant received only one format.

#### Blinding

Researchers involved in the randomization were not involved in the allocation of participants. Researchers who sorted out the formats in random order were different from those who distributed the surveys to the participants. The participants were blinded to study design and randomization. They were asked to participate in a survey about the presentation of health information and monitored while they were taking the survey. The questionnaires had the same first page, regardless of the trial group, and distributors (physicians and nurses in family practices for consumers or course teachers for students) were asked to distribute them to the next participant from the top of the survey package they received. Students completed the questionnaires before the start of their lectures, monitored by their course teacher; consumers took the survey in the waiting room, before their physicians’ appointment, and were monitored by a nurse.

#### Sample size

Sample size calculation was made using the MedCalc sample size calculator with an alpha of 0.05 and 80% power. We calculated the sample size based on knowledge score data from previous research [16] (standard deviation (*SD*)_1_ = 1.7, *SD*_2_ = 2.4, scale range from 0 to 10). To detect a difference of 20%, between average scores in knowledge among different format groups (infographics and scientific summary), we calculated that we would need 18 participants in each group (36 in total).

### Data analysis

All data collected was anonymous and stored on a server at the University of Split School of Medicine. All statistical analyses were performed using JASP v.0.9.0.0 (JASP Team, 2018, Amsterdam, Netherlands). Participants who did not complete the survey were excluded from analysis. Gender, level of education, sources of health information and Internet sources were presented as frequencies and percentages. Numeracy scores were presented as median values with interquartile range.

Comprehension scores (Trials 1 & 2), perceived effectiveness of treatment (Trials 1 & 2), desire that treatment is prescribed by their family physician (Trial 1), readiness to use the treatment (Trial 1) and preference for health information presentation (Trial 2) are presented as means with 95% confidence intervals. The differences between different framing groups were tested by using Bayesian t-test for independent samples. Considering that all participants were reading information in both numerical formats (Trial 2), the differences between the groups were tested with Bayesian repeated measures analysis of variance (frequencies vs percentages) with participant sample group (biomedical university students’ vs consumers) as between subject factor.

## Results

### Participant characteristics

**Fig. 1:**
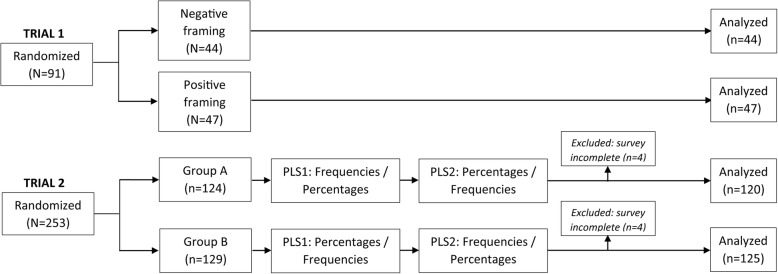
Flowchart of participants in both trials

**Trial 1.** In total, 91 participants (71% women) were enrolled in the trial, with no dropouts (Fig. 1). The most frequently reported health information source was the Internet, followed by the family physician (Table 1). Very few participants reported that they searched international websites or read research articles (Table 1).

**Table 1:** Sample characteristics in two randomized trials of summary format testing

Trials	Trial 1. Framing		Trial 2. Numerical presentation	
Variables	Negative (*n* = 44)	Positive (*n* = 47)	Group A (*n* = 120)	Group B (***n*** = 125)
**Biomedical students (%)**	44 (100)	47 (100)	52 (44.1)	43 (46.7)
**Women (%)**	32 (72.2)	33 (70.2)	75 (62.5)	83 (66.4)
**Age (Md, IQR)**	19 (19.0 to 19.8)	19 (19.0 to 20.0)	28.5 (21.0 to 47.5)	33.0 (22.0 to 55.0)
**Education (%):**				
Elementary	0	0	1 (0.8)	2 (1.6)
High school	0	0	22 (18.3)	33 (26.4)
Currently enrolled in university	44 (100)	47 (100)	55 (45.8)	47 (37.6)
College graduate	0	0	8 (6.7)	24 (19.2)
University graduate	0	0	33 (27.5)	19 (19.2)
PhD	0	0	1 (0.8)	0
**Information sources (%)**^**a**^				
Internet	27 (61.4)	34 (72.3)	90 (75.0)	95 (76.0)
Family and friends	16 (36.4)	17 (36.2)	39 (32.5)	54 (43.2)
Books	12 (27.3)	11 (23.4)	48 (40.0)	42 (33.6)
Family doctor	31 (70.5)	29 (61.7)	78 (65.0)	85 (68.0)
**Internet sources (%)**^**a**^				
First page of search results	17 (38.6)	16 (34.0)	40 (33.3)	58 (46.4)
Online forums	19 (43.2)	19 (40.4)	38 (31.7)	43 (34.4)
Hospital websites	9 (20.5)	19 (40.4)	20 (16.7)	18 (14.4)
Local specialized websites	13 (29.5)	14 (29.8)	30 (25.4)	32 (34.8)
International specialized articles	5 (11.4)	11 (23.4)	23 (19.2)	13 (10.4)
Research articles	4 (9.1)	8 (17.0)	30 (25.0)	19 (15.2)
Email to physicians on Internet websites	1 (2.3)	4 (8.5)	4 (3.3)	1 (0.8)
**Numeracy preference item**^**b**^**(Md, IQR)**			0.0 (0.0 to 1.0)	0.0 (−2.3 to 1.5)
**Objective numeracy (Md, IQR)**^**c**^	5.0 (4.0–5.0)	5.0 (4.0–5.0)	3.0 (2.0 to 4.0)	3.0 (2.0 to 4.0)

*Md* median; *IQR* interquartile range

^a^Multiple entries allowed

^b^One item scale, ranging from −4 (indicating absolute preference towards words in presentation of health information) to + 4 (indicating absolute preference towards numbers in presentation of health information)

^c^The scale was calculated as the sum of correct answers, range from 0 to 5

**Trial 2.** In total, 245 participants completed the survey (Fig. 1), of which 67% were women, and most of them had completed at least high school (Table 1). The two most frequent information sources were the Internet and the family physician (Table 1); most participants relied on the first page of search results, online forums or local specialized websites (Table 1).

### Trial 1: positive vs. negative framing of results

We found no difference between the groups that read a positively or negatively framed PLS in their assessment of the effectiveness of the investigated treatment, their desire that the treatment be offered by their family physician, their readiness to use the described treatment, as well as the number of correct answers to questions regarding the content of the PLS (Table 2).

**Table 2:** Perceived effectiveness, desire for prescription and readiness to use the described treatment between groups that read positively and negatively framed Cochrane PLSs among a student population (*N* = 91)

Variables	Framing (mean, 95% CI)		Mean difference (95% CI)	BF_**10**_^a^
	Negative (*n* = 44)	Positive (*n* = 47)		
Perceived effectiveness of treatment (score 3–30)	17.3 (15.8 to 18.8)	15.8 (14.6 to 17.0)	1.53 (−0.33 to 3.39)	1.31 × 10^− 4^
Desire that treatment be prescribed by family doctor (score 3–30)	16.1 (14.8 to 17.3)	15.4 (14.2 to 16.6)	0.69 (−1.00 to 2.37)	0.036
Readiness to use the treatment (score 3–30)	17.1 (15.8 to 18.4)	16.6 (15.2 to 18.0)	0.52 (−1.39 to 2.43)	0.035
Comprehension (score 0–12)	8.6 (8.0 to 9.3)	9.2 (8.6 to 9.7)	−0.51 (−1.43 to 0.35)	1.81 × 10^− 4^

Comprehension score, perceived effectiveness of treatment, desire that treatment be prescribed by the family doctor and readiness to use the treatment were calculated as the sum of correct answers for three PLSs. CI – confidence interval

^a^Bayesian t-test for independent samples

### Trial 2: numerical presentation

There were no significant differences in the comprehension of CSR findings between groups when they had to transform percentages to frequencies and the other way around (Table 3). Participants in both groups scored higher on the second PLS compared to the first (Table 3). Subgroup analysis showed that biomedical university students scored significantly higher compared to consumers in the comprehension of both the treatment benefits and side effects (Table 4). Moreover, no difference was found between biomedical university students and consumers in their preference for the format of the presentation and perceived effectiveness of the described treatment (Table 4).

**Table 3:** Participants’ comprehension of Cochrane PLSs between groups A and B

	Mean (95% CI)						
	Group A (***n*** = 120)		Group B ***(n*** = 125)				
**Variables**	**PLS 1**	**PLS 2**	**PLS 1**	**PLS 2**	**BF**_**10**_^**a**^	**BF**_**10**_^**b**^	**BF**_**10**_^c^
Comprehension: Results (score 0–2)	1.41 (1.27 to 1.55)	1.62 (1.50 to 1.73)	1.63 (1.52 to 1.71)	1.66 (1.56 to 1.75)	1.06	0.78	0.62
Comprehension: Side effects (score 0–2)	1.18 (1.05 to 1.30)	1.14 (1.01 to 1.26)	1.53 (1.40 to 1.66)	1.50 (1.38 to 1.63)	**1.78 × 10**^**8**^	0.14	0.13

Comprehension score is calculated as the sum of correct answers to questions in one PLS focused on benefits or side effects of a treatment. Each PLS had 4 questions, two focusing on benefits and two on side effects. CI – confidence interval, PLS – plain language summary

^a^Bayesian repeated measures ANOVA comparison of PLS 1 and PLS 2

^b^Bayesian repeated measures ANOVA, between subject effects analysis; Group

^c^Bayesian repeated measures ANOVA interaction of effects of PLS and Group

**Table 4:** Consumers’ and biomedical students’ answers on comprehension, perceived effectiveness of the described treatment and preference for health information presentation in Cochrane PLSs (*N* = 245)

	Mean (95% CI)						
	Consumers (*n* = 150)		Biomedical students (*n* = 95)				
**Variable**	**PLS 1**	**PLS 2**	**PLS 1**	**PLS 2**	**BF**_**10**_^**a**^	**BF**_**10**_^**b**^	**BF**_**10**_^**c**^
Comprehension: Results (score 0–2)	1.43 (1.31 to 1.55)	1.59 (1.50 to 1.69)	1.66 (1.53 to 1.80)	1.71 (1.59 to 1.82)	1.07	2.43	0.26
Comprehension: Side effects (score 0–2)	0.93 (0.83 to 1.04)	1.35 (1.23 to 1.49)	1.51 (1.38 to 1.63)	1.77 (1.65 to 1.89)	**1.80 × 10**^**8**^	**3.15 × 10**^**8**^	0.45
Perceived effectiveness of treatment (score 1–10)	4.53 (4.12 to 4.96)	4.93 (4.48 to 5.34)	4.84 (4.44 to 5.24)	5.35 (4.97 to 5.73)	**77.86**	0.50	0.17
Preference for health information presentation (score 1–10)	6.04 (5.65 to 6.52)	6.06 (5.60 to 6.53)	5.47 (5.03 to 5.92)	5.78 (5.39 to 6.16)	0.45	0.62	0.26

Comprehension score is calculated as the sum of correct answers to questions in a PLS focused on the benefits or side effects of treatment. Each PLS had 4 questions, two focusing on benefits and two on side effects. *CI* confidence interval, *PLS* plain language summary

^a^Repeated measures ANOVA comparison of PLS 1 and PLS 2

^b^Repeated measures analysis, between subject effects analysis Sample

^c^Repeated measures ANOVA interaction of effects of PLSs and Sample

## Discussion

Our study showed no differences in readers’ perceived effectiveness and readiness to use the described treatment when the health information in a CSR PLS was framed positively or negatively, or when the results were presented as frequencies vs percentages.

Our conclusions must be interpreted in view of several limitations. We structured, shortened and edited the tone of the PLSs used in the research, which may not be the case with every PLS, so our results can only be applied to well written and structured PLSs. Also, it is very likely that the majority of our participants did not suffer from the conditions described in the summaries, which may have affected their responses. We have tried to eliminate this bias by stating in the questionnaire that participants should answer questions as if they suffered from the described condition.

In Trial 1, the participants were first-year medical students who study medicine in a 6-year program, in which their first year is devoted to the basic sciences; based on their curriculum these students did not yet acquire advanced medical and statistical knowledge so they could be considered lay readers of PLSs. In Trial 2, we found significant differences between biomedical university students and consumers in their comprehension of benefits and side effects of the described treatment. Students who participated in Trial 2 attended senior years of biomedical university training, compared to participants from Trial 1. However, it should be noted that in Trial 2, both types of numerical presentation (frequencies and percentages) were present in the same PLS. That rarely happens when reading PLSs outside the research setting, because the presentation of results remains mostly consistent within a single PLS. We do not have information on how many consumers refused to participate in Trial 2 because the trials were performed in distant family practices. Therefore, the interpretation of results must take into account that consumers who were motivated or had potentially greater knowledge about health evidence may have volunteered for the trial.

Difficulties with the comprehension of numerical information in a health context have been recognized as a problem a long time ago, and different approaches have been attempted to improve it [17]. There is evidence that numeracy plays an important role in decision making in medicine, separately from health literacy [18], and that numeracy of health professionals presents a separate issue to consider by developers of health messages when formatting the information for lay or expert populations. In Trial 2, we found that biomedical university students scored significantly higher compared to consumers both on the comprehension of treatment benefits and side effects. These results could be explained by factors that influence health literacy in general, like age and education [19], but also by higher exposure to health-related information and formats.

We found no difference in numerical comprehension when the results in a PLS were presented as percentages or natural frequencies or when they were framed in a positive or negative direction. Participants in Trial 2 consistently had higher scores on comprehension for the second PLS compared to the those in Trial 1, regardless of the format. This could indicate that greater exposure to PLSs, or other evidence summary formats, prepares a reader for better understanding of information that follows. Familiarity with the structure of the PLS could also be a helpful factor.

A systematic review on the framing of health messages for consumers [8] found that framing has no effect on the understanding of health information. This study is, to our knowledge, the first to test the framing of health information in the context of CSR PLSs. Our results expand upon the previous findings related to plain language health messages in different contexts [8]. As there was no influence on the consumers’ understanding, we believe that the framing of the results should be left to the discretion of the authors of CSR PLSs.

Also, because most participants reported that they use information sources that are convenient and easily accessible (first pages of web searches, family physician), relevant and quality information should be made available to them. Because the confidence in health information seeking in older populations is often low [20], health care research organizations should cooperate with national policy makers to create formats for information translation that are consistent and readable to the lay population, for which some examples already exist [21, 22], and governments should enforce policies which would make those formats easily accessible to the lay population. The impact of those efforts should be measured, in order to assess the size of effect health literacy and numeracy have, both on an economical scale [13], and on healthcare, especially in shared decision making [23]. Translation of high-quality content about health from English to other languages is expected to foster dissemination among lay audience. Cochrane Croatia has been translating CSR PLSs into Croatian language since 2013 [24], but a recent survey conducted among 1000 patients in ten family physician practices across Croatia indicated that few patients have read those translated Cochrane summaries [25]. Therefore, along with the effort to create high-quality health-related content for consumers, it is also necessary to invest more effort to promote such content.

## Conclusions

Numerical presentation and framing seem to have no effect on the understanding of health information in CSR PLSs among biomedical university students and consumers. Further research should aim to assess different formats of information translation in order to improve understanding of health information, to improve populations’ health literacy and numeracy, and ultimately to contribute to better population health outcomes.

## Supplementary information

**Additional file 1 **Supplemental file legends: English translations of the modified Cochrane systematic review plain language summaries (CSR PLSs). **Supplement A**: Positive frame of numerical presentation in CSR PLSs. **Supplement B**: Negative frame of numerical presentation in CSR PLSs. **Supplement C**: Format A of numerical presentation in CSR PLSs. **Supplement D**: Format B of numerical presentation in CSR PLSs.

## Data Availability

The datasets during and/or analysed during the current study are available from the corresponding author on reasonable request.

## Abbreviations

CR: Cochrane review
PLS: Plain language summary
PLEACS: Plain Language Expectations for Authors of Cochrane Summaries

## References

[1] Kurtzman ET, Greene J (2016). Effective presentation of health care performance information for consumer decision making: a systematic review. Patient Educ Couns.

[2] Rosenbaum S, Oxman AD, Lewin S, Glenton C, Opiyo N, Sheppard S (2018). User and producer-friendly formatting of Cochrane Reviews.

[3] Jelicic Kadic A, Fidahic M, Vujcic M, Saric F, Propadalo I, Marelja I (2016). Cochrane plain language summaries are highly heterogeneous with low adherence to the standards. BMC Med Res Methodol.

[4] Reyna VF, Nelson WL, Han PK, Dieckmann NF (2009). How numeracy influences risk comprehension and medical decision making. Psychol Bull.

[5] Chen Y, Feeley TH (2014). Numeracy, information seeking, and self-efficacy in managing health: an analysis using the 2007 health information National Trends Survey (HINTS). Health Commun.

[6] Perneger TV, Agoritsas T (2011). Doctors and patients’ susceptibility to framing Bias: a randomized trial. J Gen Intern Med.

[7] Cochrane community. Editorial and Publishing Policy Resource: Plain Language Expectations for Authors of Cochrane Summaries (PLEACS). [cited August 2019]. Available from: https://community.cochrane.org/editorial-and-publishing-policy-resource/cochrane-review-development/standards-cochrane-reviews/pleacs.

[8] Akl EA, Oxman AD, Herrin J, Vist GE, Terrenato I, Sperati F (2011). Framing of health information messages. Cochrane Database Syst Rev.

[9] Derry S, Wiffen PJ, Moore RA, Bendtsen L (2015). Ibuprofen for acute treatment of episodic tension-type headache in adults. Cochrane Database Syst Rev.

[10] Richards BL, Whittle SL, Buchbinder R (2012). Muscle relaxants for pain management in rheumatoid arthritis. Cochrane Database Syst Rev.

[11] Page MJ, Green S, Kramer S, Johnston RV, McBain B, Chau M (2014). Manual therapy and exercise for adhesive capsulitis (frozen shoulder). Cochrane Database Syst Rev.

[12] Santos J, Alarcão J, Fareleira F, Vaz-Carneiro A, Costa J (2015). Tapentadol for chronic musculoskeletal pain in adults. Cochrane Database Syst Rev.

[13] Veys L, Derry S, Moore RA (2016). Ketoprofen for episodic tension-type headache in adults. Cochrane Database Syst Rev.

[14] IBM Watson Developer Cloud. Tone Analyzer. (Available at:) https://tone-analyzer-demo.mybluemix.net/ (Accessed 30 Aug 2018).

[15] Peng J, Li H, Miao D, Feng X (2013). Five different types of framing effects in medical situation: a preliminary exploration. Iran Red Crescent Med J.

[16] Buljan I, Malički M, Wager E, Puljak L, Hren D, Kellie F (2018). No difference in knowledge obtained from infographic or plain language summary of a Cochrane systematic review: three randomized controlled trials. J Clin Epidemiol.

[17] Berkman ND, Sheridan SL, Donahue KE, Halpern DJ, Crotty K (2011). Low health literacy and health outcomes: an updated systematic review. Ann Intern Med.

[18] Malloy-Weir LJ, Schwartz L, Yost J, McKibbon KA (2016). Empirical relationships between numeracy and treatment decision making: a scoping review of the literature. Patient Educ Couns.

[19] Dukic N, Blecich AA, Cerovic LJ. Economic implications of insufficient health literacy. The 6th international conference “the changing economic landscape: issues, implications and policy options. May 30th - June 1st 2013; Pula, Croatia.

[20] Magsamen-Conrad K, Dillon JM, Billotte Verhoff C, Faulkner SL (2019). Online health-information seeking among older populations: family influences and the role of the medical professional. Health Commun.

[21] Magsamen-Conrad K, Wang F, Tetteh D, Lee Y-I. Using technology adoption theory and a lifespan approach to develop a theoretical framework for eHealth literacy: extending UTAUT. Health Commun. 2019:1–12. https://www.ncbi.nlm.nih.gov/pubmed/?term=Using+technology+adoption+theory+and+a+lifespan+approach+to+develop+a+theoretical+framework+for+eHealth+literacy%3A+extending+UTAUT. 10.1080/10410236.2019.1641395 31328567

[22] Mosconi P, Antes G, Barbareschi G, Burls A, Demotes-Mainard J, Chalmers I (2016). A European multi-language initiative to make the general population aware of independent clinical research: the European communication on research awareness need project. Trials..

[23] Goggins KM, Wallston KA, Nwosu S, Schildcrout JS, Castel L, Kripalani S (2014). Health Literacy, Numeracy, and Other Characteristics Associated With Hospitalized Patients' Preferences for Involvement in Decision Making. J Health Commun.

[24] Puljak L (2016). Using social media for knowledge translation, promotion of evidence-based medicine and high-quality information on health. J Evidence-Based Med.

[25] Nejašmić D, Miošić I, Vrdoljak D, Permozer Hajdarović S, Tomičić M, Gmajnić R (2017). Awareness and use of evidence-based medicine information among patients in Croatia: a nation-wide cross-sectional study. Croat Med J.

