# Supplementary material for: Framing the numerical findings of Cochrane plain language summaries: two randomized controlled trials
Source: BMC Med Res Methodol. 2020 May 6;20:101. doi: 10.1186/s12874-020-00990-4 (PMC7201737; doi:10.1186/s12874-020-00990-4)
Supplement: Supplementary file 1 — Additional file 1 Supplemental file legends: English translations of the modified Cochrane systematic review plain language summaries (CSR PLSs). Supplement A: Positive frame of numerical presentation in CSR PLSs. Supplement B: Negative frame of numerical presentation in CSR PLSs. Supplement C: Format A of numerical presentation in CSR PLSs. Supplement D: Format B of numerical presentation in CSR PLSs. [file 12874_2020_990_MOESM1_ESM.docx]

**Supplement A: Positive frame**


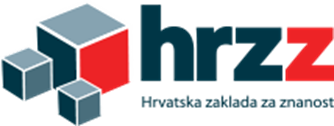

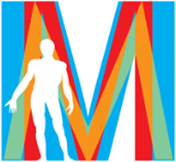


Projekt ProHealth

**Sveučilište u Splitu**

**Medicinski fakultet**

**Universitas Studiorum**

**Spalatensis**

**Facultas Medica**

**Questionnaire about health information**

Dear participant, the questionnaire before you is a part of the research project “Professionalism in Health: ProHealth” financed by Croatian Science Foundation. In this research we want to determine the optimal format of health information presentation. Your responses will be fully anonymized (gender and age data cannot reveal your identity) and will be used for research purposes only. In this questionnaire, your task will be to read brief descriptions of scientific research and answer questions about them. Continuing with this survey you give your consent to participate in the study.

**Demographic data:**

**Gender M F**

**Age**: _____________________ (In years)

**Education degree:**

a) Elementary school degree

b) High school degree

c) Currently enrolled in college/university

d) College degree

e) University degree

f) PhD degree

**Which sources do you rely on when you search for health information (Choose everything that applies to you):**

a) Internet

b) Family and friends

c) Books

d) Family doctor

e) Something else (please describe): ______________________________________-

**If you have chosen Internet as the source of health information, please answer the following question:** Which sources on the Internet do you rely on when you search for health information (Choose everything that applies to you):

a) I usually read anything that pops out first on an Internet search engine after I enter the terms of interest

b) I read Internet forums to obtain the information I search for

c) I browse he hospital websites or websites of specialized health institutions

d) I browse domestic health related websites (e.g. PlivaMed)

e) I browse international health related websites (e.g. Cochrane.org)

f) I search for research articles in scientific databases

g) I write an email to physicians available on Internet portals (e.g. Where is the evidence, cybermed.hr)

**Please read the text and answer the questions on the next page**

**Text 1:**

**Ibuprofen for acute treatment of episodic tension‐type headache in adults**

**What is it about?**

Ibuprofen is a commonly‐used painkiller available without prescription in most parts of the world. The usual dose is 400 mg taken by mouth.

**Why is it important?**

Frequent episodic tension‐type headache are described as headache that occurs between one and 14 headaches per month. Episodic tension‐type headaches are a serious condition because they stop people concentrating and working properly. When a headache occurs, the pain usually goes away over time.

**Which evidence did we find?**

In preparing this Cochrane systematic review, we searched the literature in January 2015 and found 12 studies. A total of 1800 participants were included in studies that compared ibuprofen 400 mg and placebo. Absence of headache two hours after taking medicine was considered as the result showing that the medicine was effective. Headache was gone after 2 hours in **23 out of 100** participants taking ibuprofen 400 mg, and in **16 out of 100** taking placebo.

**What is the quality of evidence?**

The studies identified in this review included only the persons who suffer from tension-type headaches, which is important to keep in mind when interpreting the results. The problems identified in retrieved studies involve the type of people chosen for the studies, and the way study outcomes are reported. This limits the usefulness of the results, especially for people who just have an occasional headache.

**Please answer the questions about the text (circle a single answer that you consider correct).**

**1. The conclusion of this systematic review is:**

A) Ibuprofen is more effective than placebo in eliminating episodic tension‐type headache.

B) Placebo is more effective than ibuprofen in eliminating episodic tension‐type headache.

C) There is no difference between ibuprofen and placebo in eliminating episodic tension‐type headache.

D) It is not clear whether there is difference between ibuprofen and placebo in eliminating episodic tension‐type headache.

**2. The effectiveness of the drug was defined in this systematic review as:**

A) Reduction in pain in the whole body

B) Ending of headache right after taking the medicine

C) Ending of headache within two hours after taking the medicine

D) Ending of headache forever

**3. These results are applicable to:**

A) All people with headache

B) All persons who are sick

C) Persons with episodic tension‐type headache

D) All persons except those with head injury

**4. Which statement is NOT correct?**

A) Episodic tension‐type headaches occur 1-14 times a month.

B) Episodic tension‐type headaches stop people concentrated.

C) Episodic tension‐type headaches are caused exclusively by stress.

D) Pain caused by episodic tension‐type headaches usually go away over time.

**In the following three questions, there are no correct answers. We ask for your opinion about the statements. Please circle the number that most closely corresponds to your opinion, on a scale from 1 to 10.**

**5. I think that ibuprofen is effective in treating tension-type headaches.**

| **Completely disagree** |  |  |  |  |  |  |  |  | **Completely agree** |
| --- | --- | --- | --- | --- | --- | --- | --- | --- | --- |
| 1 | 2 | 3 | 4 | 5 | 6 | 7 | 8 | 9 | 10 |

**6. I think that ibuprofen should be prescribed for treatment of tension-type headaches by my family medicine doctor.**

| **Completely disagree** |  |  |  |  |  |  |  |  | **Completely agree** |
| --- | --- | --- | --- | --- | --- | --- | --- | --- | --- |
| 1 | 2 | 3 | 4 | 5 | 6 | 7 | 8 | 9 | 10 |

**7. I would take ibuprofen in case that I have tension-type headache.**

| **Completely disagree** |  |  |  |  |  |  |  |  | **Completely agree** |
| --- | --- | --- | --- | --- | --- | --- | --- | --- | --- |
| 1 | 2 | 3 | 4 | 5 | 6 | 7 | 8 | 9 | 10 |

**Please read the text and answer the questions on the next page**

**Text 2:**

**Muscle relaxants for pain management in rheumatoid arthritis**

**What is it about?**

This summary of a Cochrane review presents the results from research about the effectiveness of muscle relaxants on pain in patients with rheumatoid arthritis.

**Why is it important?**

Rheumatoid arthritis is a condition in which your own immune system, which normally fights infection, attacks the lining of your joints. This makes your joints swollen, stiff, and painful. The small joints of your hands and feet are usually affected first. There is no cure for rheumatoid arthritis at present, so the treatments aim to relieve pain and stiffness and improve your ability to move.

Muscle relaxants are drugs that reduce muscle spasm or prevent increased muscle tone.

**Which evidence did we find?**

The effectiveness of muscle relaxants on pain reduction was measured at 24 hours and at one to two weeks. In both cases, it was shown that there were no differences in reducing pain between a muscle relaxant and the placebo. However, **52 out 100** persons who took muscle relaxants had side events, while **3 out 100 persons** who took placebo had side events.

**What is the quality of evidence?**

This systematic review shows that in persons with rheumatoid arthritis muscle relaxants probably do not decrease pain if the effect is measured within 24 hours or one to two weeks after taking the medication. Possible side events include fatigue, nausea, headaches, blurred vision, dry mouth, disturbance of sexual function, vertigo and constipation. Rare side events include suicidal thoughts, liver inflammation and decreased number of white blood cells.

**Please answer the questions about the text (circle a single answer that you consider correct).**

**1. The conclusion of this systematic review is:**

A) Muscle relaxants do not reduce pain in rheumatoid arthritis more than placebo.

B) Muscle relaxants reduce pain in rheumatoid arthritis more than placebo.

C) There is no difference between muscle relaxants and placebo in reducing pain in rheumatoid arthritis.

D) The difference between muscle relaxants and placebo in reducing pain in rheumatoid arthritis is not clear.

**2. The consequence of rheumatoid arthritis is NOT:**

A) Muscle inflammation

B) Joint swelling

C) Complete destruction of the immune system

D) Headache

**3. The side event that is NOT related to the use of muscle relaxants is:**

A) Headache

B) Nausea

C) Hair loss

D) Blurred vision

**4. The aim of this systematic review was to:**

A) Explore what muscle relaxants are

B) Explore what rheumatoid arthritis is

C) Explore whether muscle relaxants decrease pain in rheumatoid arthritis

D) Explore how strong the pain is in rheumatoid arthritis

**In the following three questions, there are no correct answers. We ask for your opinion about the statements. Please circle the number that most closely corresponds to your opinion, on a scale from 1 to 10.**

**5. I think that muscle relaxants are effective in treating pain in rheumatoid arthritis.**

| **Completely disagree** |  |  |  |  |  |  |  |  | **Completely agree** |
| --- | --- | --- | --- | --- | --- | --- | --- | --- | --- |
| 1 | 2 | 3 | 4 | 5 | 6 | 7 | 8 | 9 | 10 |

**6. I think that muscle relaxants should be prescribed for treatment of pain in rheumatoid arthritis by my family medicine doctor.**

| **Completely disagree** |  |  |  |  |  |  |  |  | **Completely agree** |
| --- | --- | --- | --- | --- | --- | --- | --- | --- | --- |
| 1 | 2 | 3 | 4 | 5 | 6 | 7 | 8 | 9 | 10 |

**7. I would like that muscle relaxants are used in treating pain in rheumatoid arthritis if I or someone close to me (grandmother, grandfather or other family members) had the disease**

| **Completely disagree** |  |  |  |  |  |  |  |  | **Completely agree** |
| --- | --- | --- | --- | --- | --- | --- | --- | --- | --- |
| 1 | 2 | 3 | 4 | 5 | 6 | 7 | 8 | 9 | 10 |

**Please read the text and answer the questions on the next page**

**Text 3:**

**Manual therapy and exercise for frozen shoulder**

**What is it about?**

The aim of this Cochrane systematic review was to see whether manual therapy and exercise are effective in people with frozen shoulder.

**Why is it important?**

“Frozen“ shoulder is a common cause of shoulder pain and stiffness. The pain and stiffness can last up to two to three years before spontaneously going away, and in the early stages it can be very painful.

Manual therapy comprises movement of the joints and other structures by a healthcare professional (e.g. physiotherapist). Exercise includes any purposeful movement of a joint, muscle contraction or prescribed activity. The aims of both treatments are to relieve pain, increase joint range and improve function

**Which evidence did we find?**

After searching for all relevant studies published up to May 2013, we included 32 trials in the systematic review, with a total of 1836 participants. The studied compared the effectiveness of manual therapy and exercise with a shoulder block – glucocorticoid (a steroid that reduces inflammation) injection into the shoulder. After six weeks of therapy, **46 out of 100** participants rated the manual therapy and exercise as more successfully in comparison to **77 out of 100** participants who were treated with glucocorticoid injection. Side effects were mild, like short-term pain after therapy, and there was no difference between the groups.

**What is the quality of evidence?**

Best available evidence points that manual therapy and exercise are not more effective than glucocorticoid injection for the treatment of frozen shoulder. No studies compared the effect of manual therapy and exercise with placebo or no treatment or other forms of treatment. Further studies are need to compare manual therapy and exercise to other forms of treatments.

**Please answer the questions about the text (circle a single answer that you consider correct).**

**1. The conclusion of this systematic review is:**

A) Manual therapy and exercise are more effective than glucocorticoid injection in treating frozen shoulder.

B) Glucocorticoid injection is more effective than manual therapy and exercise in treating frozen shoulder.

C) There is no difference between manual therapy with exercise and glucocorticoids in treating frozen shoulder.

D) It is not clear whether there is difference between manual therapy with exercise and glucocorticoids in treating frozen shoulder.

**2. The symptom of frozen shoulder is:**

A) Redness and itching

B) Increased temperature and swelling

C) Pain and stiffness

D) Tingling

**3. The statement that is NOT correct for manual therapy and exercise:**

A) It aims to improve joint function

B) It includes purposeful movements of joint and other structures performed by a physiotherapist

C) It aims to relieve pain

D) It means immobilization of joints and other structures

**4. “Shoulder block” designates:**

A) Loss of shoulder joint function

B) Glucocorticoid (a steroid that reduces inflammation) injection into the shoulder

C) Special form of physical therapy

D) Injection of ibuprofen in the shoulder

**In the following three questions, there are no correct answers. We ask for your opinion about the statements. Please circle the number that most closely corresponds to your opinion, on a scale from 1 to 10.**

**5. I think manual therapy and exercise are effective in treating frozen shoulder.**

| **Completely disagree** |  |  |  |  |  |  |  |  | **Completely agree** |
| --- | --- | --- | --- | --- | --- | --- | --- | --- | --- |
| 1 | 2 | 3 | 4 | 5 | 6 | 7 | 8 | 9 | 10 |

**6. I think manual therapy and exercise should be prescribed for treating frozen shoulder by my family medicine doctor.**

| **Completely disagree** |  |  |  |  |  |  |  |  | **Completely agree** |
| --- | --- | --- | --- | --- | --- | --- | --- | --- | --- |
| 1 | 2 | 3 | 4 | 5 | 6 | 7 | 8 | 9 | 10 |

**7. I would use manual therapy and exercise if I had frozen shoulder.**

| **Completely disagree** |  |  |  |  |  |  |  |  | **Completely agree** |
| --- | --- | --- | --- | --- | --- | --- | --- | --- | --- |
| 1 | 2 | 3 | 4 | 5 | 6 | 7 | 8 | 9 | 10 |

**In the following five questions there is only one correct answer. Please choose only one answer you think it is correct.**

1. A medical study will randomly assign people so they are equally likely to get medicine A or medicine B. If there are 300 people in the study, about how many are expected to get medicine A?

a. 100 people

b. 150 people

c. 200 people

d. 250 people

2. Older age and smoking both increase the risk of a heart attack over time. David is now 50 years old and smokes. His risk of a heart attack in the next 10 years is 10%. If he continues to smoke which of the following could be his risk of a heart attack over the next 20 years?

a. 5%

b. 10%

c. 30%

d. 100%

3. James starts a new blood pressure medicine. The chance of a serious side effect is 0.5%. If 1000 people take this medicine, about how many would be expected to have a serious side effect?

a. 1 person

b. 5 people

c. 50 people

d. 500 people

4. The PSA (Prostate Specific Antigen) is a blood test that can be used to screen for prostate cancer. However, 30% of men who have an abnormal test result will turn out not to have cancer. John has an abnormal test result. What is the chance that John has prostate cancer?

a. 0%

b. 30%

c. 70%

d. 100%

5. Rebecca is treated for stage 2 breast cancer. The chance that the cancer will come back is 10% over 10 years. If Rebecca takes a new medicine, this chance will decrease by 30%. If 100 women like Rebecca take this medicine, how many are now expected to have breast cancer come back within 10 years?

a. 3 out of 100 women

b. 7 out of 100 women

c. 10 out of 100 women

d. 30 out of 100 women

THE END!

Thank you for your participation!

**Supplement B: Negative frame**


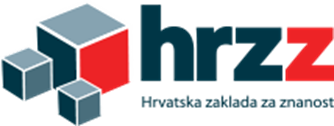

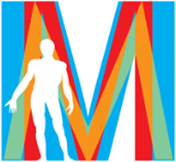


Projekt ProHealth

**Sveučilište u Splitu**

**Medicinski fakultet**

**Universitas Studiorum**

**Spalatensis**

**Facultas Medica**

**Questionnaire about health information**

Dear participant, the questionnaire before you is a part of the research project “Professionalism in Health: ProHealth” financed by Croatian Science Foundation. In this research we want to determine the optimal format of health information presentation. Your responses will be fully anonymized (gender and age data cannot reveal your identity) and will be used for research purposes only. In this questionnaire, your task will be to read brief descriptions of scientific research and answer questions about them. Continuing with this survey you give your consent to participate in the study.

**Demographic data:**

**Gender M F**

**Age**: _____________________ (In years)

**Education degree:**

a) Elementary school degree

b) High school degree

c) Currently enrolled in college/university

d) College degree

e) University degree

f) PhD degree

**Which sources do you rely on when you search for health information (Choose everything that applies to you):**

a) Internet

b) Family and friends

c) Books

d) Family doctor

e) Something else (please describe): ______________________________________-

**If you have chosen Internet as the source of health information, please answer the following question:** Which sources on the Internet do you rely on when you search for health information (Choose everything that applies to you):

a) I usually read anything that pops out first on an Internet search engine after I enter the terms of interest

b) I read Internet forums to obtain the information I search for

c) I browse he hospital websites or websites of specialized health institutions

d) I browse domestic health related websites (e.g. PlivaMed)

e) I browse international health related websites (e.g. Cochrane.org)

f) I search for research articles in scientific databases

g) I write an email to physicians available on Internet portals (e.g. Where is the evidence, cybermed.hr)

**Please read the text and answer the questions on the next page**

**Text 1:**

**Ibuprofen for acute treatment of episodic tension‐type headache in adults**

**What is it about?**

Ibuprofen is a commonly‐used painkiller available without prescription in most parts of the world. The usual dose is 400 mg taken by mouth.

**Why is it important?**

Frequent episodic tension‐type headache are described as headache that occurs between one and 14 headaches per month. Episodic tension‐type headaches are a serious condition because they stop people concentrating and working properly. When a headache occurs, the pain usually goes away over time.

**Which evidence did we find?**

In preparing this Cochrane systematic review, we searched the literature in January 2015 and found 12 studies. A total of 1800 participants were included in studies that compared ibuprofen 400 mg and placebo. Absence of headache two hours after taking medicine was considered as the result showing that the medicine was effective. Headache was not gone after 2 hours in **77 out of 100** participants taking ibuprofen 400 mg, and in **84 out of 100** taking placebo.

**What is the quality of evidence?**

The studies identified in this review included only the persons who suffer from tension-type headaches, which is important to keep in mind when interpreting the results. The problems identified in retrieved studies involve the type of people chosen for the studies, and the way study outcomes are reported. This limits the usefulness of the results, especially for people who just have an occasional headache.

**Please answer the questions about the text (circle a single answer that you consider correct).**

**1. The conclusion of this systematic review is:**

A) Ibuprofen is more effective than placebo in eliminating episodic tension‐type headache.

B) Placebo is more effective than ibuprofen in eliminating episodic tension‐type headache.

C) There is no difference between ibuprofen and placebo in eliminating episodic tension‐type headache.

D) It is not clear whether there is difference between ibuprofen and placebo in eliminating episodic tension‐type headache.

**2. The effectiveness of the drug was defined in this systematic review as:**

A) Reduction in pain in the whole body

B) Ending of headache right after taking the medicine

C) Ending of headache within two hours after taking the medicine

D) Ending of headache forever

**3. These results are applicable to:**

A) All people with headache

B) All persons who are sick

C) Persons with episodic tension‐type headache

D) All persons except those with head injury

**4. Which statement is NOT correct?**

A) Episodic tension‐type headaches occur 1-14 times a month.

B) Episodic tension‐type headaches stop people concentrated.

C) Episodic tension‐type headaches are caused exclusively by stress.

D) Pain caused by episodic tension‐type headaches usually go away over time.

**In the following three questions, there are no correct answers. We ask for your opinion about the statements. Please circle the number that most closely corresponds to your opinion, on a scale from 1 to 10.**

**5. I think that ibuprofen is effective in treating tension-type headaches.**

| **Completely disagree** |  |  |  |  |  |  |  |  | **Completely agree** |
| --- | --- | --- | --- | --- | --- | --- | --- | --- | --- |
| 1 | 2 | 3 | 4 | 5 | 6 | 7 | 8 | 9 | 10 |

**6. I think that ibuprofen should be prescribed for treatment of tension-type headaches by my family medicine doctor.**

| **Completely disagree** |  |  |  |  |  |  |  |  | **Completely agree** |
| --- | --- | --- | --- | --- | --- | --- | --- | --- | --- |
| 1 | 2 | 3 | 4 | 5 | 6 | 7 | 8 | 9 | 10 |

**7. I would take ibuprofen in case that I have tension-type headache.**

| **Completely disagree** |  |  |  |  |  |  |  |  | **Completely agree** |
| --- | --- | --- | --- | --- | --- | --- | --- | --- | --- |
| 1 | 2 | 3 | 4 | 5 | 6 | 7 | 8 | 9 | 10 |

**Please read the text and answer the questions on the next page**

**Text 2:**

**Muscle relaxants for pain management in rheumatoid arthritis**

**What is it about?**

This summary of a Cochrane review presents the results from research about the effectiveness of muscle relaxants on pain in patients with rheumatoid arthritis.

**Why is it important?**

Rheumatoid arthritis is a condition in which your own immune system, which normally fights infection, attacks the lining of your joints. This makes your joints swollen, stiff, and painful. The small joints of your hands and feet are usually affected first. There is no cure for rheumatoid arthritis at present, so the treatments aim to relieve pain and stiffness and improve your ability to move.

Muscle relaxants are drugs that reduce muscle spasm or prevent increased muscle tone.

**Which evidence did we find?**

The effectiveness of muscle relaxants on pain reduction was measured at 24 hours and at one to two weeks. In both cases, it was shown that there were no differences in reducing pain between a muscle relaxant and the placebo. However, **48 out 100** persons who took muscle relaxants did not have side events, while **97 out 100 persons** who took placebo did not have side events.

**What is the quality of evidence?**

This systematic review shows that in persons with rheumatoid arthritis muscle relaxants probably do not decrease pain if the effect is measured within 24 hours or one to two weeks after taking the medication. Possible side events include fatigue, nausea, headaches, blurred vision, dry mouth, disturbance of sexual function, vertigo and constipation. Rare side events include suicidal thoughts, liver inflammation and decreased number of white blood cells.

**Please answer the questions about the text (circle a single answer that you consider correct).**

**1. The conclusion of this systematic review is:**

A) Muscle relaxants do not reduce pain in rheumatoid arthritis more than placebo.

B) Muscle relaxants reduce pain in rheumatoid arthritis more than placebo.

C) There is no difference between muscle relaxants and placebo in reducing pain in rheumatoid arthritis.

D) The difference between muscle relaxants and placebo in reducing pain in rheumatoid arthritis is not clear.

**2. The consequence of rheumatoid arthritis is NOT:**

A) Muscle inflammation

B) Joint swelling

C) Complete destruction of the immune system

D) Headache

**3. The side event that is NOT related to the use of muscle relaxants is:**

A) Headache

B) Nausea

C) Hair loss

D) Blurred vision

**4. The aim of this systematic review was to:**

A) Explore what muscle relaxants are

B) Explore what rheumatoid arthritis is

C) Explore whether muscle relaxants decrease pain in rheumatoid arthritis

D) Explore how strong the pain is in rheumatoid arthritis

**In the following three questions, there are no correct answers. We ask for your opinion about the statements. Please circle the number that most closely corresponds to your opinion, on a scale from 1 to 10.**

**5. I think that muscle relaxants are effective in treating pain in rheumatoid arthritis.**

| **Completely disagree** |  |  |  |  |  |  |  |  | **Completely agree** |
| --- | --- | --- | --- | --- | --- | --- | --- | --- | --- |
| 1 | 2 | 3 | 4 | 5 | 6 | 7 | 8 | 9 | 10 |

**6. I think that muscle relaxants should be prescribed for treatment of pain in rheumatoid arthritis by my family medicine doctor.**

| **Completely disagree** |  |  |  |  |  |  |  |  | **Completely agree** |
| --- | --- | --- | --- | --- | --- | --- | --- | --- | --- |
| 1 | 2 | 3 | 4 | 5 | 6 | 7 | 8 | 9 | 10 |

**7. I would like that muscle relaxants are used in treating pain in rheumatoid arthritis if I or someone close to me (grandmother, grandfather or other family members) had the disease**

| **Completely disagree** |  |  |  |  |  |  |  |  | **Completely agree** |
| --- | --- | --- | --- | --- | --- | --- | --- | --- | --- |
| 1 | 2 | 3 | 4 | 5 | 6 | 7 | 8 | 9 | 10 |

**Please read the text and answer the questions on the next page**

**Text 3:**

**Manual therapy and exercise for frozen shoulder**

**What is it about?**

The aim of this Cochrane systematic review was to see whether manual therapy and exercise are effective in people with frozen shoulder.

**Why is it important?**

“Frozen“ shoulder is a common cause of shoulder pain and stiffness. The pain and stiffness can last up to two to three years before spontaneously going away, and in the early stages it can be very painful.

Manual therapy comprises movement of the joints and other structures by a healthcare professional (e.g. physiotherapist). Exercise includes any purposeful movement of a joint, muscle contraction or prescribed activity. The aims of both treatments are to relieve pain, increase joint range and improve function

**Which evidence did we find?**

After searching for all relevant studies published up to May 2013, we included 32 trials in the systematic review, with a total of 1836 participants. The studied compared the effectiveness of manual therapy and exercise with a shoulder block – glucocorticoid (a steroid that reduces inflammation) injection into the shoulder. After six weeks of therapy, **54 out of 100** participants rated the manual therapy and exercise as less successful in comparison to **23 out of 100** participants who were treated with glucocorticoid injection. Side effects were mild, like short-term pain after therapy, and there was no difference between the groups.

**What is the quality of evidence?**

Best available evidence points that manual therapy and exercise are not more effective than glucocorticoid injection for the treatment of frozen shoulder. No studies compared the effect of manual therapy and exercise with placebo or no treatment or other forms of treatment. Further studies are need to compare manual therapy and exercise to other forms of treatments.

**Please answer the questions about the text (circle a single answer that you consider correct).**

**1. The conclusion of this systematic review is:**

A) Manual therapy and exercise are more effective than glucocorticoid injection in treating frozen shoulder.

B) Glucocorticoid injection is more effective than manual therapy and exercise in treating frozen shoulder.

C) There is no difference between manual therapy with exercise and glucocorticoids in treating frozen shoulder.

D) It is not clear whether there is difference between manual therapy with exercise and glucocorticoids in treating frozen shoulder.

**2. The symptom of frozen shoulder is:**

A) Redness and itching

B) Increased temperature and swelling

C) Pain and stiffness

D) Tingling

**3. The statement that is NOT correct for manual therapy and exercise:**

A) It aims to improve joint function

B) It includes purposeful movements of joint and other structures performed by a physiotherapist

C) It aims to relieve pain

D) It means immobilization of joints and other structures

**4. “Shoulder block” designates:**

A) Loss of shoulder joint function

B) Glucocorticoid (a steroid that reduces inflammation) injection into the shoulder

C) Special form of physical therapy

D) Injection of ibuprofen in the shoulder

**In the following three questions, there are no correct answers. We ask for your opinion about the statements. Please circle the number that most closely corresponds to your opinion, on a scale from 1 to 10.**

**5. I think manual therapy and exercise are effective in treating frozen shoulder.**

| **Completely disagree** |  |  |  |  |  |  |  |  | **Completely agree** |
| --- | --- | --- | --- | --- | --- | --- | --- | --- | --- |
| 1 | 2 | 3 | 4 | 5 | 6 | 7 | 8 | 9 | 10 |

**6. I think manual therapy and exercise should be prescribed for treating frozen shoulder by my family medicine doctor.**

| **Completely disagree** |  |  |  |  |  |  |  |  | **Completely agree** |
| --- | --- | --- | --- | --- | --- | --- | --- | --- | --- |
| 1 | 2 | 3 | 4 | 5 | 6 | 7 | 8 | 9 | 10 |

**7. I would use manual therapy and exercise if I had frozen shoulder.**

| **Completely disagree** |  |  |  |  |  |  |  |  | **Completely agree** |
| --- | --- | --- | --- | --- | --- | --- | --- | --- | --- |
| 1 | 2 | 3 | 4 | 5 | 6 | 7 | 8 | 9 | 10 |

**In the following five questions there is only one correct answer. Please choose only one answer you think it is correct.**

1. A medical study will randomly assign people so they are equally likely to get medicine A or medicine B. If there are 300 people in the study, about how many are expected to get medicine A?

a. 100 people

b. 150 people

c. 200 people

d. 250 people

2. Older age and smoking both increase the risk of a heart attack over time. David is now 50 years old and smokes. His risk of a heart attack in the next 10 years is 10%. If he continues to smoke which of the following could be his risk of a heart attack over the next 20 years?

a. 5%

b. 10%

c. 30%

d. 100%

3. James starts a new blood pressure medicine. The chance of a serious side effect is 0.5%. If 1000 people take this medicine, about how many would be expected to have a serious side effect?

a. 1 person

b. 5 people

c. 50 people

d. 500 people

4. The PSA (Prostate Specific Antigen) is a blood test that can be used to screen for prostate cancer. However, 30% of men who have an abnormal test result will turn out not to have cancer. John has an abnormal test result. What is the chance that John has prostate cancer?

a. 0%

b. 30%

c. 70%

d. 100%

5. Rebecca is treated for stage 2 breast cancer. The chance that the cancer will come back is 10% over 10 years. If Rebecca takes a new medicine, this chance will decrease by 30%. If 100 women like Rebecca take this medicine, how many are now expected to have breast cancer come back within 10 years?

a. 3 out of 100 women

b. 7 out of 100 women

c. 10 out of 100 women

d. 30 out of 100 women

THE END!

Thank you for your participation!

**Supplement C: Format A**


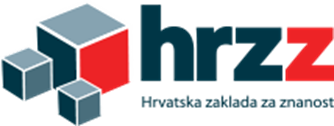

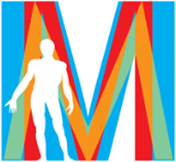


Projekt ProHealth

**Sveučilište u Splitu**

**Medicinski fakultet**

**Universitas Studiorum**

**Spalatensis**

**Facultas Medica**

**Questionnaire about health information**

Dear participant, the questionnaire before you is a part of the research project “Professionalism in Health: ProHealth” financed by Croatian Science Foundation. In this research we want to determine the optimal format of health information presentation. Your responses will be fully anonymized (gender and age data cannot reveal your identity) and will be used for research purposes only. In this questionnaire, your task will be to read brief descriptions of scientific research and answer questions about them. Continuing with this survey you give your consent to participate in the study.

**Demographic data:**

**Gender M F**

**Age**: _____________________ (In years)

**Education degree:**

a) Elementary school degree

b) High school degree

c) Currently enrolled in college/university

d) College degree

e) University degree

f) PhD degree

**Which sources do you rely on when you search for health information (Choose everything that applies to you):**

a) Internet

b) Family and friends

c) Books

d) Family doctor

e) Something else (please describe): ______________________________________-

**If you have chosen Internet as the source of health information, please answer the following question:** Which sources on the Internet do you rely on when you search for health information (Choose everything that applies to you):

a) I usually read anything that pops out first on an Internet search engine after I enter the terms of interest

b) I read Internet forums to obtain the information I search for

c) I browse he hospital websites or websites of specialized health institutions

d) I browse domestic health related websites (e.g. PlivaMed)

e) I browse international health related websites (e.g. Cochrane.org)

f) I search for research articles in scientific databases

g) I write an email to physicians available on Internet portals (e.g. Where is the evidence, cybermed.hr)

**Tapentadol for chronic musculoskeletal pain in adults**

**What is it about?**

Pain in bones, joints, and muscles is very common and can often be persistent. It is expected that 50% of the general population will suffer from this type of pain for at least three months or longer during their lifetime. This condition is called chronic musculoskeletal pain. Opioids are a type of strong pain‐killer drug that are used to treat people that have moderate‐to‐severe chronic pain. People taking these drugs frequently have side effects, including severe side effects.

**What did the researchers do?**

This Cochrane review aimed to assess the effectiveness (reduction in pain intensity) and safety of tapentadol (a new opioid) in moderate‐to‐severe chronic musculoskeletal pain, in comparison to placebo (a pretend medicine) and other drugs that work for such pain.

We performed a literature search in March 2014 for studies that compared tapentadol with placebo or other drugs in adults with musculoskeletal pain.

**Which evidence did the researchers find?**

We found four studies comparing tapentadol with placebo or oxycodone (another opioid) in 4094 adults.

There was moderate‐quality evidence that 3 out of 10 people treated with tapentadol had pain reduction (responded to the treatment) while the pain was reduced in only 2 out of 10 people who took placebo or oxycodone.

There was also moderate‐quality evidence that tapentadol‐treated people were at a higher risk of withdrawal from the trial due to side effects in comparison to placebo (20% tapentadol‐treated people and 10% placebo‐treated people). For oxycodone‐treated people, 40% withdrew due to side effects. Constipation, nausea and vomiting, and itching (pruritus) were less with tapentadol than with oxycodone but there was no difference in fatigue, insomnia, sleepiness (somnolence), and headache.

**What is the quality of evidence found?**

The overall clinical benefit of tapentadol in moderate‐to‐severe chronic musculoskeletal pain found in clinical trials was relatively small (a common conclusion found in all opioids trials for chronic pain). Further studies are needed to find out which people with chronic musculoskeletal pain would benefit the most from this new opioid.

**Choose the correct answer:**

1. How many people had pain reduction after taking tapentadol?

a) 30% b) 20% c) 3%

2. What is the difference in tapentadol effectiveness in comparison to oxycodone or placebo, i.e., how many more people had benefit from taking tapentadol in comparison to oxycodone of placebo?

a) 10% b) 1% c) 0.1%

3. What is the difference in the number of people who had side-effects because of tapentadol in comparison to the number of people who took oxycodone or placebo, i.e., how many more people had side effects when they took tapentadol in comparison to oxycodone or placebo?

a) 1 out of 100 b) 1 out of 10 c) 2 out of 10

4. What is the percentage of people taking tapentadol who withdrew from the trial?

a) 40 out 100 b) 25 out of 100 c) 4 out of 100

**In the following three questions, there are no correct answers. We ask for your opinion about the statements. Please circle the number that most closely corresponds to your opinion, on a scale from 1 to 10.**

**5. I think that tapentadol is effective in reducing chronic musculoskeletal pain.**

| **Completely disagree** |  | |  | |  | |  | |  | |  | |  | | **Completely agree** | |
| --- | --- | --- | --- | --- | --- | --- | --- | --- | --- | --- | --- | --- | --- | --- | --- | --- |
| 1 | 2 | 3 | | 4 | | 5 | | 6 | | 7 | | 8 | | 9 | | 10 |

**6. This is appropriate way of presenting health information.**

| **Completely disagree** |  | |  | |  | |  | |  | |  | |  | | **Completely agree** | |
| --- | --- | --- | --- | --- | --- | --- | --- | --- | --- | --- | --- | --- | --- | --- | --- | --- |
| 1 | 2 | 3 | | 4 | | 5 | | 6 | | 7 | | 8 | | 9 | | 10 |

**7. Please mark on the scale which is you preferred way of presenting health information (-4 means that you prefer only words, and +4 means that you prefer only numbers)**

| **I prefer words only** |  |  |  |  |  |  |  | **I prefer numbers only** |
| --- | --- | --- | --- | --- | --- | --- | --- | --- |
| -4 | -3 | -2 | -1 | 0 | +1 | +2 | +3 | +4 |

**8. If you think you need more information so that you can decide on whether tapentadol was effective, what would that be? Please write your opinion below.**

**Ketoprofen for treatment of acute episodic tension‐type headache in adults**

**What is it about?**

People with frequent episodic tension‐type headache have between 2 and 14 headaches days every month. Tension‐type headache stops people concentrating and working properly, and results in much disability. When headaches do occur, they get better over time, even without treatment. Ketoprofen is a commonly used painkiller, available by prescription in most parts of the world but without prescription (over‐the‐counter) in some. The usual dose is 25 mg or 50 mg taken by mouth. The aim of this systematic review was to assess whether ketoprofen is effective in treating frequent episodic tension‐type headache in adults.

**What did the researchers do?**

In May 2016, we searched the medical literature and found four studies involving 1253 participants looking at ketoprofen for frequent episodic tension‐type headache. Only a fraction of the participants was involved in comparisons between ketoprofen 25 mg and placebo (a dummy tablet). Results were reported two hours after taking the medicine or placebo. The International Headache Society recommends the outcome of being pain‐free two hours after taking a medicine, but other outcomes are also suggested. Few studies reported these recommended outcomes, so there was limited information to analyse for some outcomes.

**Which evidence did the researchers find?**

The outcome of being pain‐free at two hours was reported by 27% people taking ketoprofen 25 mg, and in 16% people taking placebo. This means that only 11% people benefited because of ketoprofen.

The outcome of being pain‐free or having only mild pain at two hours was reported by 66% people taking ketoprofen 25 mg, and in 38% people taking placebo. This means that 28% people benefited because of ketoprofen 25 mg.

Side-effects were reported by 14 out of 100 people taking ketoprofen, which was slightly more than with placebo (7 out of 100 people). Most side effects were mild or moderate in intensity. No side effects were serious.

Ketoprofen was not different from paracetamol 1000 mg for any measure of headache relief, but was associated with more side events.

**What is the quality of evidence?**

The quality of the evidence for being pain‐free at 2 hours was low quality, and for having mild pain at 2 hours was moderate quality. Moderate quality evidence means that we are reasonably confident about the results. Low quality evidence means that we are not very certain about the results and they could change with more information.

**Choose the correct answer:**

1. In how many people ketoprofen was effective in being headache-free at 2 hours after medicine taking?

a) 27 out of 100 b) 25% out of 100 c) 16 out of 100

2. How many people had benefit from taking ketoprofen for reducing pain in comparison to those who took placebo?

a) 16 out of 100 b) 28 out of 100 c) 38 out of 100

3. How many people had side effects related to taking ketoprofen?

a) 14% b) 66% c) 25%

4. What is the difference in the number of people who had side effects because of ketoprofen in comparison to the number of people who had side effects because of placebo?

a) 28% b) 7% c) 14%

**In the following three questions, there are no correct answers. We ask for your opinion about the statements. Please circle the number that most closely corresponds to your opinion, on a scale from 1 to 10.**

**5. I think that ketoprofen is effective in reducing pain caused by tension-type headache.**

| **Completely disagree** |  | |  | |  | |  | |  | |  | |  | | **Completely agree** | |
| --- | --- | --- | --- | --- | --- | --- | --- | --- | --- | --- | --- | --- | --- | --- | --- | --- |
| 1 | 2 | 3 | | 4 | | 5 | | 6 | | 7 | | 8 | | 9 | | 10 |

**6. This is appropriate way of presenting health information.**

| **Completely disagree** |  | |  | |  | |  | |  | |  | |  | | **Completely agree** | |
| --- | --- | --- | --- | --- | --- | --- | --- | --- | --- | --- | --- | --- | --- | --- | --- | --- |
| 1 | 2 | 3 | | 4 | | 5 | | 6 | | 7 | | 8 | | 9 | | 10 |

**7. Please mark on the scale which is you preferred way of presenting health information (-4 means that you prefer only words, and +4 means that you prefer only numbers)**

| **I prefer words only** |  |  |  |  |  |  |  | **I prefer numbers only** |
| --- | --- | --- | --- | --- | --- | --- | --- | --- |
| -4 | -3 | -2 | -1 | 0 | +1 | +2 | +3 | +4 |

**8. If you think you need more information so that you can decide on whether ketoprofen was effective, what would that be? Please, write your opinion below.**

**Answer:___________________________________________________________________.**

**In the following five questions there is only one correct answer. Please choose only one answer you think it is correct.**

1. A medical study will randomly assign people so they are equally likely to get medicine A or medicine B. If there are 300 people in the study, about how many are expected to get medicine A?

a. 100 people

b. 150 people

c. 200 people

d. 250 people

2. Older age and smoking both increase the risk of a heart attack over time. David is now 50 years old and smokes. His risk of a heart attack in the next 10 years is 10%. If he continues to smoke which of the following could be his risk of a heart attack over the next 20 years?

a. 5%

b. 10%

c. 30%

d. 100%

3. James starts a new blood pressure medicine. The chance of a serious side effect is 0.5%. If 1000 people take this medicine, about how many would be expected to have a serious side effect?

a. 1 person

b. 5 people

c. 50 people

d. 500 people

4. The PSA (Prostate Specific Antigen) is a blood test that can be used to screen for prostate cancer. However, 30% of men who have an abnormal test result will turn out not to have cancer. John has an abnormal test result. What is the chance that John has prostate cancer?

a. 0%

b. 30%

c. 70%

d. 100%

5. Rebecca is treated for stage 2 breast cancer. The chance that the cancer will come back is 10% over 10 years. If Rebecca takes a new medicine, this chance will decrease by 30%. If 100 women like Rebecca take this medicine, how many are now expected to have breast cancer come back within 10 years?

a. 3 out of 100 women

b. 7 out of 100 women

c. 10 out of 100 women

d. 30 out of 100 women

THE END!

Thank you for your participation!

**Supplement D: Format B**


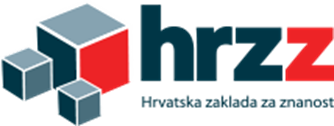

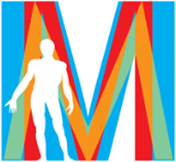


Projekt ProHealth

**Sveučilište u Splitu**

**Medicinski fakultet**

**Universitas Studiorum**

**Spalatensis**

**Facultas Medica**

**Questionnaire about health information**

Dear participant, the questionnaire before you is a part of the research project “Professionalism in Health: ProHealth” financed by Croatian Science Foundation. In this research we want to determine the optimal format of health information presentation. Your responses will be fully anonymized (gender and age data cannot reveal your identity) and will be used for research purposes only. In this questionnaire, your task will be to read brief descriptions of scientific research and answer questions about them. Continuing with this survey you give your consent to participate in the study.

**Demographic data:**

**Gender M F**

**Age**: _____________________ (In years)

**Education degree:**

a) Elementary school degree

b) High school degree

c) Currently enrolled in college/university

d) College degree

e) University degree

f) PhD degree

**Which sources do you rely on when you search for health information (Choose everything that applies to you):**

a) Internet

b) Family and friends

c) Books

d) Family doctor

e) Something else (please describe): ______________________________________-

**If you have chosen Internet as the source of health information, please answer the following question:** Which sources on the Internet do you rely on when you search for health information (Choose everything that applies to you):

a) I usually read anything that pops out first on an Internet search engine after I enter the terms of interest

b) I read Internet forums to obtain the information I search for

c) I browse he hospital websites or websites of specialized health institutions

d) I browse domestic health related websites (e.g. PlivaMed)

e) I browse international health related websites (e.g. Cochrane.org)

f) I search for research articles in scientific databases

g) I write an email to physicians available on Internet portals (e.g. Where is the evidence, cybermed.hr)

**Tapentadol for chronic musculoskeletal pain in adults**

**What is it about?**

Pain in bones, joints, and muscles is very common and can often be persistent. It is expected that 50% of the general population will suffer from this type of pain for at least three months or longer during their lifetime. This condition is called chronic musculoskeletal pain. Opioids are a type of strong pain‐killer drug that are used to treat people that have moderate‐to‐severe chronic pain. People taking these drugs frequently have side effects, including severe side effects.

**What did the researchers do?**

This Cochrane review aimed to assess the effectiveness (reduction in pain intensity) and safety of tapentadol (a new opioid) in moderate‐to‐severe chronic musculoskeletal pain, in comparison to placebo (a pretend medicine) and other drugs that work for such pain.

We performed a literature search in March 2014 for studies that compared tapentadol with placebo or other drugs in adults with musculoskeletal pain.

**Which evidence did the researchers find?**

We found four studies comparing tapentadol with placebo or oxycodone (another opioid) in 4094 adults.

There was moderate‐quality evidence that 30% of people treated with tapentadol had pain reduction (responded to the treatment) while the pain was reduced in only 20% of the people who took placebo or oxycodone.

There was also moderate‐quality evidence that tapentadol‐treated people were at a higher risk of withdrawal from the trial due to side effects in comparison to placebo (2 out of 10 tapentadol‐treated people and 1 out of 10 placebo‐treated people). For oxycodone‐treated people, 4 out of 10 withdrew due to side effects. Constipation, nausea and vomiting, and itching (pruritus) were less with tapentadol than with oxycodone but there was no difference in fatigue, insomnia, sleepiness (somnolence), and headache.

**What is the quality of evidence found?**

The overall clinical benefit of tapentadol in moderate‐to‐severe chronic musculoskeletal pain found in clinical trials was relatively small (a common conclusion found in all opioids trials for chronic pain). Further studies are needed to find out which people with chronic musculoskeletal pain would benefit the most from this new opioid.

**Choose the correct answer:**

1. How many people had pain reduction after taking tapentadol?

a) 3 out of 10 b) 2 out of 10 c) 3 out of 100

2. What is the difference in tapentadol effectiveness in comparison to oxycodone or placebo, i.e., how many more people had benefit from taking tapentadol in comparison to oxycodone of placebo?

a) 1 out of 10 b) 1 out of 100 c) 1 out of od 1000

3. What is the difference in the number of people who had side-effects because of tapentadol in comparison to the number of people who took oxycodone or placebo, i.e., how many more people had side effects when they took tapentadol in comparison to oxycodone or placebo?

a) 1% b) 10 % c) 20%

4. What is the percentage of people taking tapentadol who withdrew from the trial?

a) 40% b) 25% c) 4%

**In the following three questions, there are no correct answers. We ask for your opinion about the statements. Please circle the number that most closely corresponds to your opinion, on a scale from 1 to 10.**

**5. I think that tapentadol is effective in reducing chronic musculoskeletal pain.**

| **Completely disagree** |  | |  | |  | |  | |  | |  | |  | | **Completely agree** | |
| --- | --- | --- | --- | --- | --- | --- | --- | --- | --- | --- | --- | --- | --- | --- | --- | --- |
| 1 | 2 | 3 | | 4 | | 5 | | 6 | | 7 | | 8 | | 9 | | 10 |

**6. This is appropriate way of presenting health information.**

| **Completely disagree** |  | |  | |  | |  | |  | |  | |  | | **Completely agree** | |
| --- | --- | --- | --- | --- | --- | --- | --- | --- | --- | --- | --- | --- | --- | --- | --- | --- |
| 1 | 2 | 3 | | 4 | | 5 | | 6 | | 7 | | 8 | | 9 | | 10 |

**7. Please mark on the scale which is you preferred way of presenting health information (-4 means that you prefer only words, and +4 means that you prefer only numbers)**

| **I prefer words only** |  |  |  |  |  |  |  | **I prefer numbers only** |
| --- | --- | --- | --- | --- | --- | --- | --- | --- |
| -4 | -3 | -2 | -1 | 0 | +1 | +2 | +3 | +4 |

**8. If you think you need more information so that you can decide on whether tapentadol was effective, what would that be? Please write your opinion below.**

**Ketoprofen for treatment of acute episodic tension‐type headache in adults**

**What is it about?**

People with frequent episodic tension‐type headache have between 2 and 14 headaches days every month. Tension‐type headache stops people concentrating and working properly, and results in much disability. When headaches do occur, they get better over time, even without treatment. Ketoprofen is a commonly used painkiller, available by prescription in most parts of the world but without prescription (over‐the‐counter) in some. The usual dose is 25 mg or 50 mg taken by mouth. The aim of this systematic review was to assess whether ketoprofen is effective in treating frequent episodic tension‐type headache in adults.

**What did the researchers do?**

In May 2016, we searched the medical literature and found four studies involving 1253 participants looking at ketoprofen for frequent episodic tension‐type headache. Only a fraction of the participants was involved in comparisons between ketoprofen 25 mg and placebo (a dummy tablet). Results were reported two hours after taking the medicine or placebo. The International Headache Society recommends the outcome of being pain‐free two hours after taking a medicine, but other outcomes are also suggested. Few studies reported these recommended outcomes, so there was limited information to analyse for some outcomes.

**Which evidence did the researchers find?**

The outcome of being pain‐free at two hours was reported by 27 in 100 people taking ketoprofen 25 mg, and in 16 out of 100 people taking placebo. This means that only 11 in 100 people benefited because of ketoprofen.

The outcome of being pain‐free or having only mild pain at two hours was reported by 66 in 100 people taking ketoprofen 25 mg, and in 38 out of 100 people taking placebo. This means that 28 in 100 people benefited because of ketoprofen 25 mg.

Side-effects were reported by 15% of people taking ketoprofen, which was slightly more than with placebo (7% people). Most side effects were mild or moderate in intensity. No side effects were serious.

Ketoprofen was not different from paracetamol 1000 mg for any measure of headache relief, but was associated with more side events.

**What is the quality of evidence?**

The quality of the evidence for being pain‐free at 2 hours was low quality, and for having mild pain at 2 hours was moderate quality. Moderate quality evidence means that we are reasonably confident about the results. Low quality evidence means that we are not very certain about the results and they could change with more information.

**Choose the correct answer:**

1. In how many people ketoprofen was effective in being headache-free at 2 hours after medicine taking?

a) 27% b) 25% c) 16%

2. How many people had benefit from taking ketoprofen for reducing pain in comparison to those who took placebo?

a) 16% b) 28% c) 38%

3. How many people had side effects related to taking ketoprofen?

a) 14 od 100 b) 66 od 100 c) 25 od 100

4. What is the difference in the number of people who had side effects because of ketoprofen in comparison to the number of people who had side effects because of placebo?

a) 28 od 100 b) 7 od 100 c) 14 od 100

**In the following three questions, there are no correct answers. We ask for your opinion about the statements. Please circle the number that most closely corresponds to your opinion, on a scale from 1 to 10.**

**5. I think that ketoprofen is effective in reducing pain caused by tension-type headache.**

| **Completely disagree** |  | |  | |  | |  | |  | |  | |  | | **Completely agree** | |
| --- | --- | --- | --- | --- | --- | --- | --- | --- | --- | --- | --- | --- | --- | --- | --- | --- |
| 1 | 2 | 3 | | 4 | | 5 | | 6 | | 7 | | 8 | | 9 | | 10 |

**6. This is appropriate way of presenting health information.**

| **Completely disagree** |  | |  | |  | |  | |  | |  | |  | | **Completely agree** | |
| --- | --- | --- | --- | --- | --- | --- | --- | --- | --- | --- | --- | --- | --- | --- | --- | --- |
| 1 | 2 | 3 | | 4 | | 5 | | 6 | | 7 | | 8 | | 9 | | 10 |

**7. Please mark on the scale which is you preferred way of presenting health information (-4 means that you prefer only words, and +4 means that you prefer only numbers)**

| **I prefer words only** |  |  |  |  |  |  |  | **I prefer numbers only** |
| --- | --- | --- | --- | --- | --- | --- | --- | --- |
| -4 | -3 | -2 | -1 | 0 | +1 | +2 | +3 | +4 |

**8. If you think you need more information so that you can decide on whether ketoprofen was effective, what would that be? Please, write your opinion below.**

**Answer:___________________________________________________________________.**

**In the following five questions there is only one correct answer. Please choose only one answer you think it is correct.**

1. A medical study will randomly assign people so they are equally likely to get medicine A or medicine B. If there are 300 people in the study, about how many are expected to get medicine A?

a. 100 people

b. 150 people

c. 200 people

d. 250 people

2. Older age and smoking both increase the risk of a heart attack over time. David is now 50 years old and smokes. His risk of a heart attack in the next 10 years is 10%. If he continues to smoke which of the following could be his risk of a heart attack over the next 20 years?

a. 5%

b. 10%

c. 30%

d. 100%

3. James starts a new blood pressure medicine. The chance of a serious side effect is 0.5%. If 1000 people take this medicine, about how many would be expected to have a serious side effect?

a. 1 person

b. 5 people

c. 50 people

d. 500 people

4. The PSA (Prostate Specific Antigen) is a blood test that can be used to screen for prostate cancer. However, 30% of men who have an abnormal test result will turn out not to have cancer. John has an abnormal test result. What is the chance that John has prostate cancer?

a. 0%

b. 30%

c. 70%

d. 100%

5. Rebecca is treated for stage 2 breast cancer. The chance that the cancer will come back is 10% over 10 years. If Rebecca takes a new medicine, this chance will decrease by 30%. If 100 women like Rebecca take this medicine, how many are now expected to have breast cancer come back within 10 years?

a. 3 out of 100 women

b. 7 out of 100 women

c. 10 out of 100 women

d. 30 out of 100 women

THE END!

Thank you for your participation!
